# A Comprehensive Analysis of Liver Lipidomics Signature in Adults with Metabolic Dysfunction-Associated Steatohepatitis—A Pilot Study

**DOI:** 10.3390/ijms252313067

**Published:** 2024-12-05

**Authors:** Thomai Mouskeftara, Georgios Kalopitas, Theodoros Liapikos, Konstantinos Arvanitakis, Eleni Theocharidou, Georgios Germanidis, Helen Gika

**Affiliations:** 1 Laboratory of Forensic Medicine & Toxicology, Department of Medicine, Aristotle University of Thessaloniki, 54124 Thessaloniki, Greece; mousthom@auth.gr; 2 Division of Gastroenterology and Hepatology, 1st Department of Internal Medicine, AHEPA University Hospital, School of Medicine, Faculty of Health Sciences, Aristotle University of Thessaloniki, 54124 Thessaloniki, Greece; gekalopi@auth.gr (G.K.); arvanitak@auth.gr (K.A.); 3 Basic and Translational Research Unit, Special Unit for Biomedical Research and Education, School of Medicine, Faculty of Health Sciences, Aristotle University of Thessaloniki, 54636 Thessaloniki, Greece; 4 Laboratory of Hygiene, Social and Preventive Medicine and Medical Statistics, School of Medicine, Faculty of Health Sciences, Aristotle University of Thessaloniki, 54124 Thessaloniki, Greece; 5 Laboratory of Analytical Chemistry, Department of Chemistry, Aristotle University of Thessaloniki, 54124 Thessaloniki, Greece; theoliapikos@gmail.com; 6 2nd Department of Internal Medicine, Hippokration General Hospital, Aristotle University of Thessaloniki, 54642 Thessaloniki, Greece; eltheocharidou@hotmail.com; 7 Biomic AUTh, Center for Interdisciplinary Research and Innovation (CIRI-AUTH), Balkan Center B1.4, 10th km Thessaloniki-Thermi Rd., 57001 Thessaloniki, Greece

**Keywords:** liver biopsies, untargeted lipidomics analysis, fatty acid profile, Metabolic Dysfunction-Associated Steatohepatitis, Metabolic Dysfunction-Associated Steatotic Liver Disease

## Abstract

Metabolic Dysfunction-Associated Steatotic Liver Disease (MASLD) is the most common chronic liver disorder in Western countries, encompassing a range of conditions from steatosis to Metabolic Dysfunction-Associated Steatohepatitis (MASH), which can potentially progress to cirrhosis. Lipidomics approaches have revealed significant alterations in the hepatic lipidome associated with both steatosis and steatohepatitis, with these changes correlating with disease manifestation. While the transition from steatosis to MASH remains poorly understood, recent research indicates that both the quantity and quality of deposited lipids play a pivotal role in MASLD progression. In our study, we utilized untargeted and targeted analyses to identify intact lipids and fatty acids in liver biopsies from healthy controls and MASLD patients, categorized based on their histological findings. In total, 447 lipid species were identified, with 215 subjected to further statistical analysis. Univariate and multivariate analyses revealed alterations in triglyceride species and fatty acids, including FA 16:0, FA 16:1, FA 18:3 n6, the sum of MUFA, and the Δ9-desaturase activity ratio. This research provides insights into the connection between dysregulated lipid metabolism in the progression of MASLD, supporting previous findings. Further studies on lipid metabolism could improve risk assessment methods, particularly given the current limited understanding of the transition from steatosis to MASH.

## 1. Introduction

Metabolic Dysfunction-Associated Steatotic Liver Disease (MASLD) previously known as nonalcoholic fatty liver disease (NAFLD) is a pathological condition characterized by excessive intrahepatic fat accumulation and clinically characterized by a broad disease spectrum, encompassing Metabolic Dysfunction-Associated Steatotic Liver (MASL) and Metabolic Dysfunction-Associated Steatohepatitis (MASH) [1,2,3]. Simple steatosis with or without inflammation characterizes MASL, whereas MASH is defined by the coexistence of steatosis, inflammation and hepatocellular ballooning, which increase the risks of cirrhosis, hepatocellular cancer, and hepatic fibrosis [4,5]. Additionally, MASLD is a systemic condition linked to metabolic syndrome, type 2 diabetes, insulin resistance, and obesity [6,7,8,9]. The significant increase in these incidences, now affecting over 1 billion individuals, placing MASLD as the most prevalent cause of chronic liver conditions and a major global public health concern [10,11,12].

The initial explanation for the progression of MASLD from simple steatosis to MASH was based on the “two-hit” model. This model proposed that the initial hit was hepatic steatosis, which made the liver more sensitive to a second hit that triggered inflammatory responses and fibrosis [13]. However, recent research has revealed that a more intricate process involving multiple simultaneous “metabolic hits” is likely responsible for tissue damage, encompassing various factors and pathogenic mechanisms [14]. Insulin resistance leads to increased hepatic de novo lipogenesis, reduced inhibition of lipolysis in adipose tissue, and subsequently, an elevated fatty acid flux to the liver [15]. As most free fatty acids (FFAs) are stored in triglycerides (TG) within intracellular lipid droplets, an imbalance between the uptake, synthesis, export, and oxidation of FFA can lead to the accumulation of various lipid species [16]. This lipid deposition in the liver tissue impairs cellular functions, causing lipotoxicity characterized by mitochondrial dysfunction, endoplasmic reticulum stress, reactive oxygen species production, oxidative stress and activation of inflammatory pathways [17,18,19,20,21,22]. Consequently, this leads to hepatocellular damage, inflammation, and fibrosis [23]. Considering the crucial role of lipids in the pathophysiology of MASLD, it is essential to investigate lipidomic dysregulations, as changes in the lipidome may contribute to or be linked with the onset and progression of MASLD [24].

The field of lipidomics aims to comprehensively examine different molecular lipid species in biological systems, investigate their functions and reveal alterations in their levels within the matrix of interest (e.g., liver, plasma, adipose tissue) [25,26]. Research in the field of human and animal studies indicates that notable disturbances in the liver’s lipidome could be potential key contributors to the mechanism of disease progression towards MASH, encompassing alterations in glycerophospholipids, sphingolipids, fatty acids, and glycerolipids [27,28,29,30]. In some cases, the types of lipids associated with disease progression are debated, as is the impact of their localization [31,32]. However, the extensive results on lipidomics in MASLD in the literature complicate forming a clear understanding, as many lipid markers and their fold changes between disease states reported by different studies are inconsistent [29].

The aim of this study was to analyze in detail the lipidomic profile linked to the increasing severity of MASLD and the presence of MASH in the context of a controlled pilot study. We examined changes in liver lipidome to identify lipids potentially associated with liver disease. Using both untargeted and targeted analyses, we identified intact lipids and fatty acids in 18 liver biopsies from healthy controls and MASLD patients, categorized by their histological findings. A total of 447 lipid species from 14 major lipid subclasses were identified, with 215 undergoing further statistical analysis. Univariate and multivariate analyses showed alterations in triglyceride species and fatty acids, such as FA 16:0, FA 16:1, FA 18:3 n6, the sum of MUFA, and the Δ9–desaturase activity ratio. This research sheds light on the connection between dysregulated lipid metabolism and MASLD progression, reinforcing previous findings. Further studies on lipid metabolism could enhance risk assessment methods, particularly given the current limited understanding of the transition from steatosis to MASH. In our project, the participants fulfilled both the old and new nomenclature criteria. However, since the study population was recruited prior to the adoption of the new MASLD terminology, the previously used terms—NAFLD, NAFL and NASH—will be applied throughout the manuscript to describe the study groups.

## 2. Results

### 2.1. Baseline Characteristics of Study Population

This research project enrolled 18 individuals, including patients suspected of suffering from NAFLD and a control group. The NAFLD patients underwent a percutaneous ultrasound-guided liver biopsy, conforming to current clinical practice guidelines, and were classified as having NAFL (27.7%) or NASH (39.0%) based on the NAFLD Activity Score (NAS) evaluation as described by Kalopitas et al. [33]. The control group consisted of 33.3% of the participants, who underwent a liver biopsy after informed consent during laparoscopic cholecystectomy surgery. The demographic and clinical characteristics of the three groups are presented in Table 1. The analysis of the data revealed that gender, waist circumference, total triglycerides, and uric acid levels differed significantly between the control group and NASH patients. Additionally, histological findings and scores, including NAS, FIB-4, steatosis, inflammation, and fibrosis, were found to differ significantly between the control group and NASH patients. Furthermore, the biochemical and histological parameters AST, HOMA-IR, insulin, and ballooning were also found to differ in both comparisons between the control and NASH groups and between the NAFL-NASH groups.

### 2.2. Liver Lipid Profile in Patients with NAFLD and Controls

**Figure 1:**
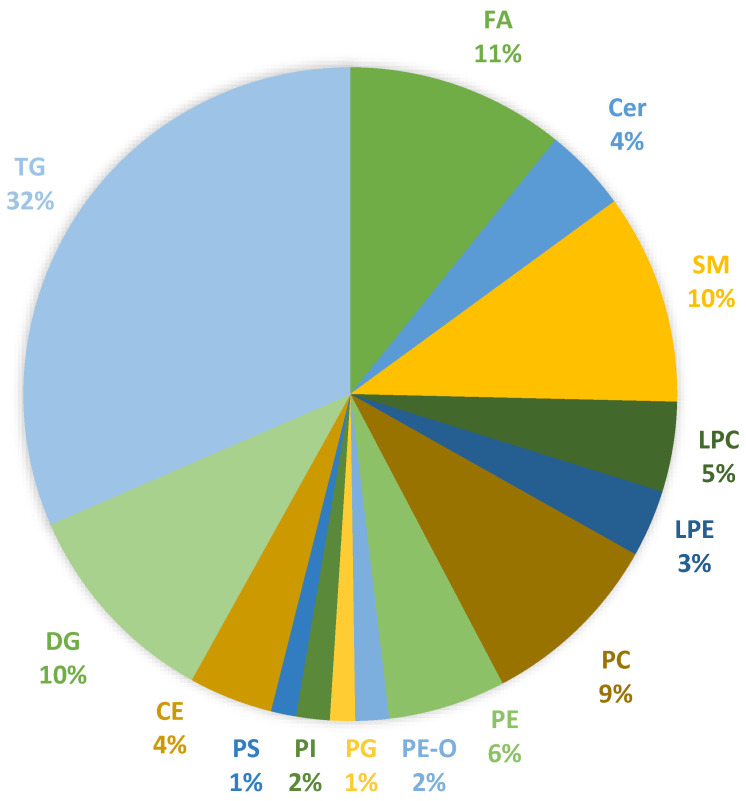
Lipid subclasses identified in liver biopsies of NAFLD patients and healthy controls based on targeted and untargeted analyses of fatty acids and esterified lipids. Abbreviations: FA: fatty acids, Cer: ceramides, SM: sphingomyelins, LPC: monoacylglycerophosphocholines, LPE: monoacylglycerophosphoethanolamines, PC: diacylglycerophosphocholines, PE: diacylglycerophosphoethanolamines, PE-O: 1-alkyl,2-acylglycerophosphoethanolamines, PG: diacylglycerophosphoglycerols, PI: diacylglycerophosphoinositols, PS: diacylglycerophosphoserines, CE: cholesterol esters, DG: diglycerides, and TG: triglycerides.

**Figure 2:**
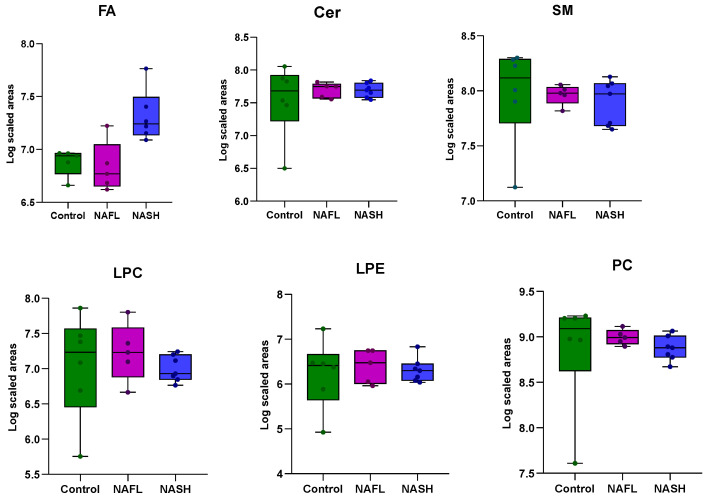

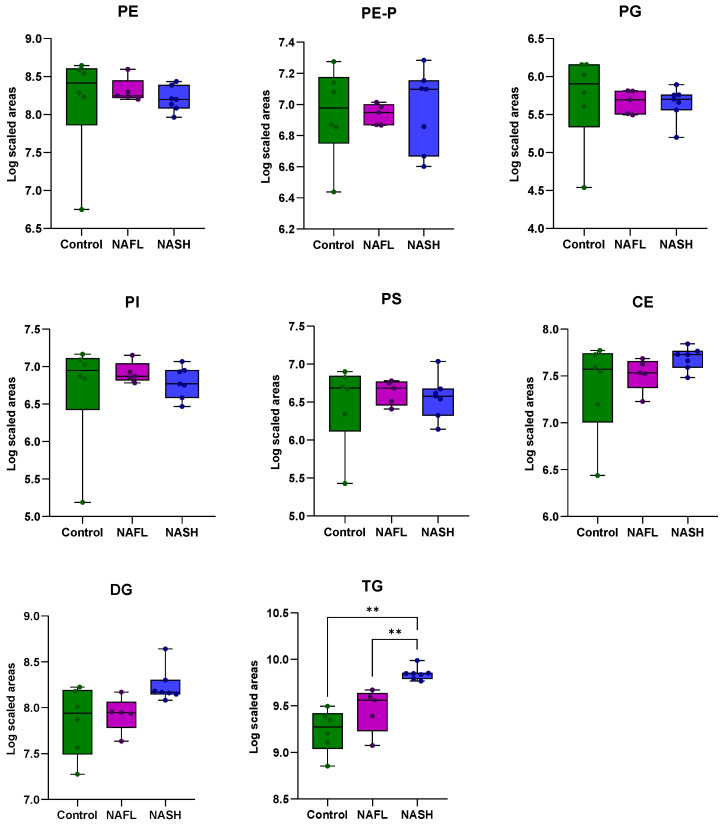
Lipid class composition (in normalized peak areas; log10 scaled) of liver biopsies in the three main groups of the study. The total abundance of each lipid class was calculated by adding up peak areas of lipid species. Boxes highlight the values between 25% and 75% quartiles; vertical lines connect minimum and maximum values. Statistically significant difference was observed in the class of TGs between controls and NASH (** *p*-value = 5.2 × 10^−6^) and in NAFL–NASH patients (** *p*-value = 4.25 × 10^−4^).

A total of 18 liver biopsy samples were analyzed, including individuals with NAFLD and healthy controls, using an untargeted lipidomic approach for the investigation of intact lipids, while a targeted GC-MS method was used for the analysis of 26 fatty acids. The untargeted lipidomic analysis identified 447 lipid species, and statistical processing was conducted on 215 lipids. The identified lipid categories included fatty acids (10.7%), sphingolipids (14.6%), glycerophospholipids (28.6%), and glycerolipids (46.1%). The diagram in Figure 1 illustrates the 14 subcategories of lipid classes detected in liver biopsy samples. Meanwhile, Figure 2 features box plots that present the composition of these 14 major lipid classes in liver biopsies across the three study groups. There was no marked difference between the total abundance of Cer, SM, LPC, LPE PC, and PE-P in disease groups compared with controls, except from a trend of slightly lower levels of PE, PG, PI, and PS in NASH patients compared to the other two groups. Additionally, higher levels of FA, CE, DG, and TG were found in NASH patients, with the difference being statistically significant only for the TG class. In Table S1, comprehensive information on the identified lipid species is provided, including annotations of molecular species, the molecular formulas, the monoisotopic masses, and retention time data.

### 2.3. LC-QTOF-MS Analysis Revealed Alterations in TG Species

**Figure 3:**
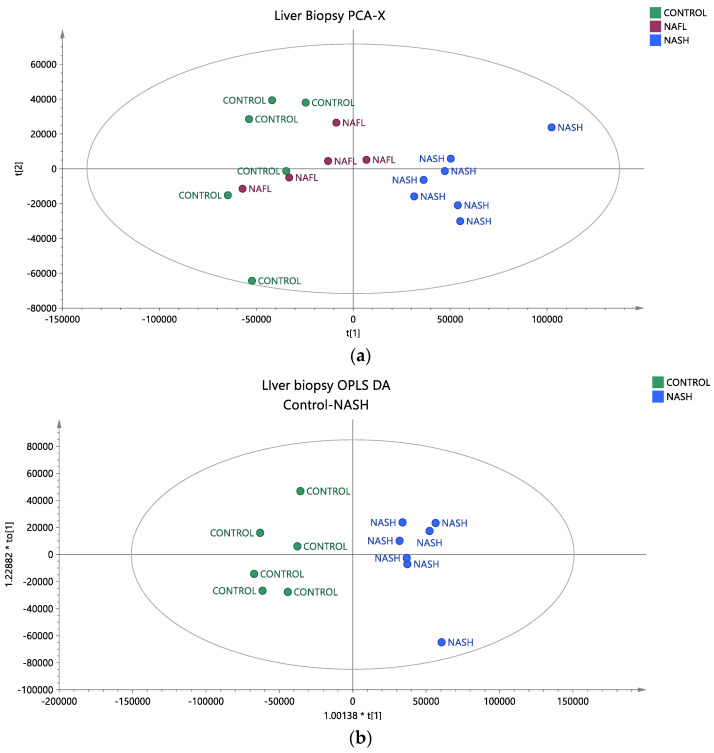

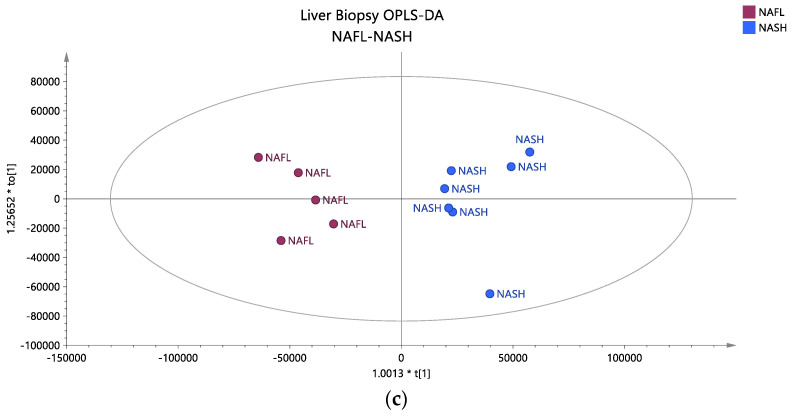
(**a**) Unsupervised PCA model depicting the studied groups in the liver biopsy, where healthy controls are depicted with green dots, patients with NAFL with purple dots and patients with NASH with blue dots (R^2^X = 0.823, Q^2^ = 0.736). (**b**) Supervised OPLS-DA model depicting the differentiation between samples of healthy controls and patients with NASH (R^2^X = 0.823, R^2^Y = 0.942, Q^2^ = 0.871, CV ANOVA *p*-value = 1.25 × 10^−3^). (**c**) Supervised OPLS-DA model depicting the differentiation between patients with NAFL and patients with NASH (R^2^X = 0.797, R^2^Y = 0.897, Q^2^ = 0.782, CV ANOVA *p*-value = 1.81 × 10^−2^). Pareto scaling was used in all models.

**Figure 4:**
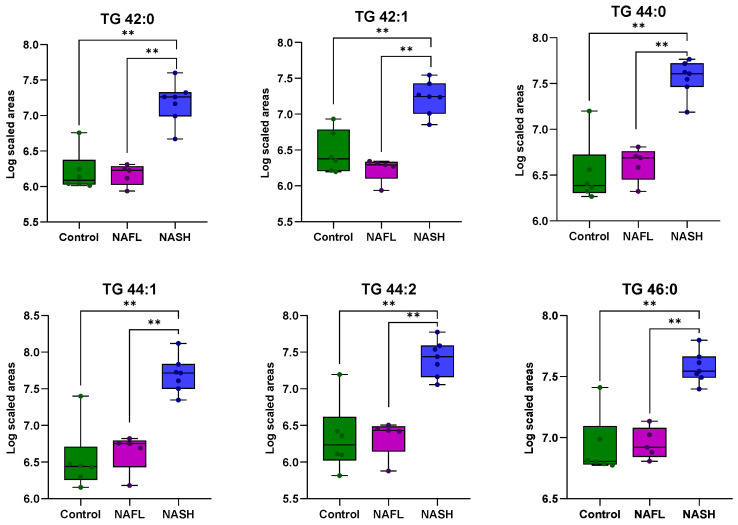

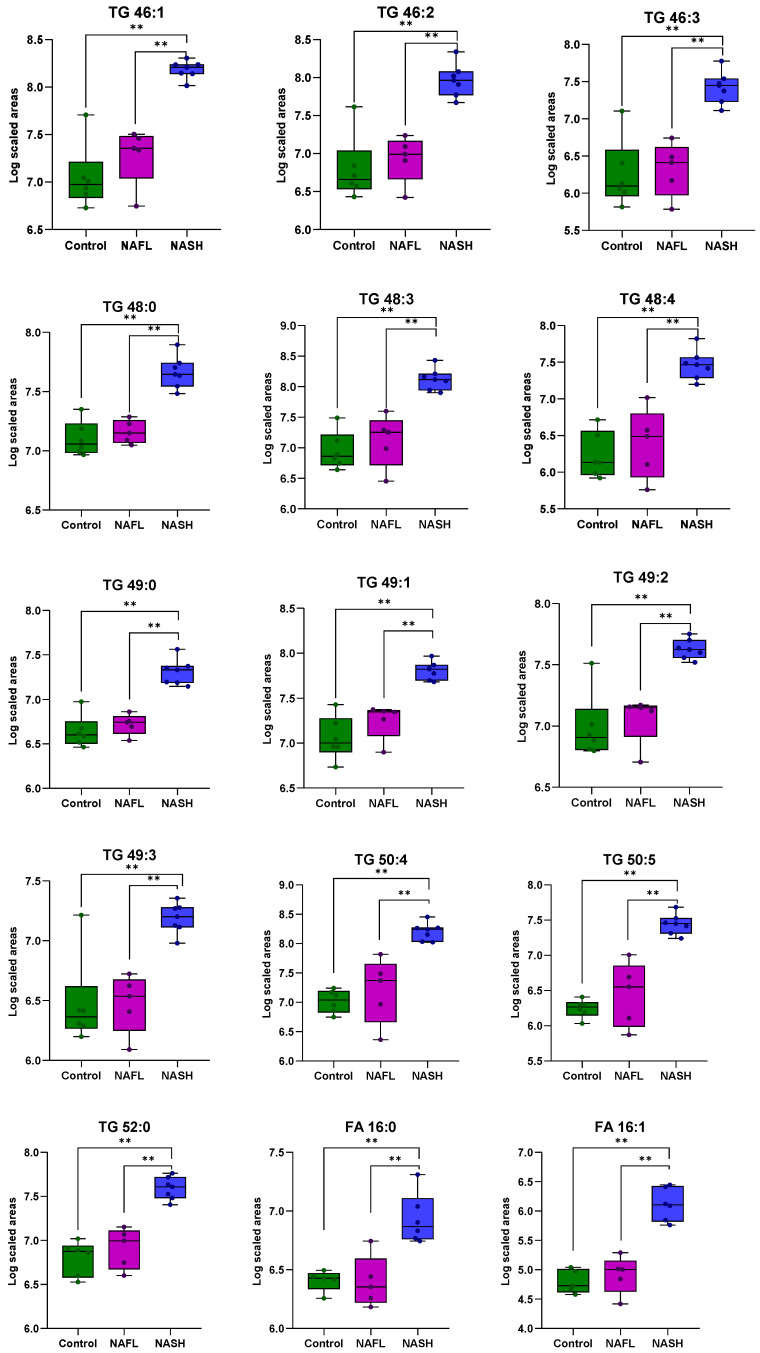

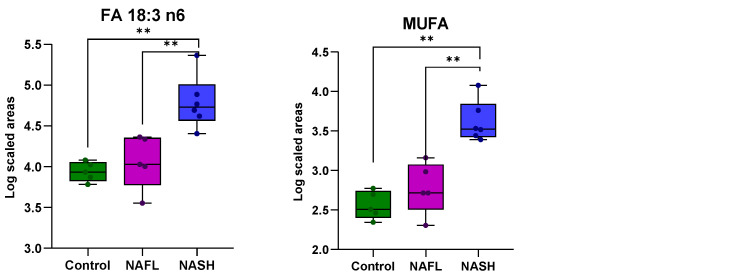
Boxplots showing the distribution of the 19 TG species, FA 16:0, FA 16:1, FA 18:3 ω6, and MUFA across the studied groups. These lipids were common to both healthy controls–NASH patients and NAFL–NASH patient comparisons. *p* values ≤ 0.05 are highlighted **.

To assess whether the liver tissue lipid profiles of individuals with NAFLD and healthy controls differ based on the stage of the disease, univariate and multivariate statistical analyses were conducted. The reliability of the analytical system was first verified by examining the QC samples. A PCA score plot, which encompassed all samples and QC samples, exhibited satisfactory analytical precision and is depicted in Figure S1. Additionally, any lipids with CV% values higher than 30% in the QC samples were excluded from further analysis. In the unsupervised PCA plots, it is evident that the samples tend to separate into distinct groups. The healthy controls (green dots) are positioned on the left side of the plot, while the patients with NASH (blue dots) occupy the right side (Figure 3a). The patients with NAFL (purple dots) are situated in the center of the plot and are close to the healthy controls. To further investigate the initial differentiation observed, a pairwise OPLS-DA analysis was conducted, which identified statistically significant lipids that differentiate healthy controls from patients with NASH and patients with NAFL from those with NASH, as shown in Figure 3b,c, respectively. All information regarding the unsupervised and supervised models, along with the CV ANOVA values for the statistical significance of the models, is provided in Table S2. Comparison of healthy controls and NASH patients revealed 38 TGs that differed, with elevated levels observed in the latter group. In the analysis of NAFL and NASH patients, 19 TGs exhibited statistically significant differences, with higher levels observed in the NASH group as well. All TGs that distinguished NAFL from NASH patients were also found to be statistically significant in the control-NAFL comparison. In Figure 4, the common TG species between the two comparisons are presented as box plots, providing information on their levels across the various groups studied. To adjust for potential confounding factors, ANCOVA analysis was performed to adjust the *p* values based on waist circumference and HOMA-IR values. In both comparisons, TGs with a *p*-value < 0.05 before and after ANCOVA analysis were considered statistically significant. All statistically significant TGs, along with their *p*-values, adjusted *p*-values*, VIP, Log2FC, and CV%, are presented in Table 2.

### 2.4. Fatty Acid Profile in Liver Samples

A total of 26 fatty acids were measured in the liver biopsy samples of NAFLD patients and healthy controls using a targeted GC-MS method. The methyl esters of these fatty acids are presented in Table S1. Given the consistent correlation between hepatic steatosis and fatty acids with varying degrees of unsaturation in their carbon chains, the levels of saturated (SFA), monounsaturated (MUFA), and polyunsaturated (PUFA) fatty acids in each group were studied. Moreover, the lipogenesis index (DNL index), elongation index (ELOG), and desaturation indices concerning the relative activity of Δ5-desaturase, Δ6-desaturase, and Δ9-desaturase were investigated. The DNL index was calculated as the ratio of palmitic acid (FA 16:0) to polyunsaturated linoleic acid (FA 18:2). The elongation index for FA 16:0 was defined as the ratio of FA 18:0 to FA 16:0. The Δ5, Δ6, and Δ9 desaturase indices were calculated as the ratios of FA 18:2/FA 20:4, FA 18:2/FA 18:3, FA 16:1/FA 16:0, and FA 18:1/FA 18:0, respectively. Univariate statistical analysis between the study groups showed differences in FA 16:0, FA 16:1, FA 18:3 n6 fatty acids, and the sum of MUFA in both comparisons, healthy controls–NASH patients and NAFL–NASH patients, as presented in Figure 4. The relative activity of Δ9-desaturase was found to be significant only when comparing healthy controls to NASH patients. In all cases, statistically significant fatty acids and ratios were found at higher levels in patients with NASH. ANCOVA analysis was performed to adjust the *p*-values based on waist circumference and HOMA-IR values. Fatty acids and ratios with a *p*-value ≤ 0.05 before and after ANCOVA analysis were considered statistically significant. Table 3 includes all statistically significant fatty acids and ratios along with their *p*-values, adjusted p* values, and Log2FC.

## 3. Discussion

In this study, we analyzed liver biopsy samples from 18 healthy control and patients suspected of having NAFLD and classified them as NAFL or NASH based on liver histology using untargeted lipidomic and targeted GC-MS analyses. A key finding of our study is that patients with NASH had a unique liver lipid profile distinct from those with NAFL and healthy controls, despite both NASH and NAFL groups having similar established metabolic risk factors [33].

Our research focused on investigating differences in lipidomes, specifically fatty acids, ceramides, sphingomyelins, (Lyso)phosphatidylethanolamines, (Lyso)phosphatidylcholines, phosphatidylinositols, phosphatidylglycerols, diglycerides, triglycerides, and cholesterol esters, to identify lipids associated with the phenotype and severity of NAFLD. We employed both untargeted and targeted lipid analysis methods to assess disease stratification using an extremely small amount of tissue (minimum quantity: 0.39 mg of liver biopsy). Initially, we compared NASH patients to healthy controls and found that 38 TGs were elevated in the NASH group. In total, 50% of these TGs had saturated and monounsaturated fatty acid chains, while the remaining 50% included at least one polyunsaturated fatty acid. When comparing NAFL to NASH patients, 19 TGs showed differences, with 63.1% containing saturated and monounsaturated fatty acids and 36.9% containing polyunsaturated fatty acids.

Prior lipidomic studies on liver tissue samples from NAFLD patients have revealed a notable increase in TG levels [34,35,36,37] containing saturated and monounsaturated fatty acids, while decreased TG levels containing polyunsaturated fatty acids, including omega-3 and omega-6 fatty acids, which were found in patients with NAFLD [37]. The study by Gorden et al. analyzed 186 lipids in liver biopsy specimens as well as 132 plasma lipids of 91 patients, revealing 48 common species [38]. Specifically, in liver tissue, 83 TG species were found to be statistically different across the studied groups as presented by their ANOVA *p* values and 35 of them were found statistically significant in our study, including the following TG species: TG 44:2, TG 44:1, TG 44:0, TG 46:3, TG 46:2, TG 46:1, TG 46:0, TG 48:4, TG 48:3, TG 48:2, TG 48:1, TG 48:0, TG 49:3, TG 49:2, TG 49:1, TG 49:0, TG 50:5, TG 50:4, TG 50:3, TG 50:2, TG 50:1, TG 50:0, TG 51:3, TG 51:2, TG 51:1, TG 52:5, TG 52:4 TG 52:3, TG 52:1, TG 52:0, TG 53:3, TG 53:2, TG 53:1, TG 54:2 and TG 54:1. In a study conducted by Chiappini et al., a comparative lipidomic analysis was performed on 61 human liver biopsies from patients with NAFL, NASH, or controls using TOF-SIMS. This led to the identification and quantification of 104 lipid species of 32 lipid classes, including five triglycerides, namely TG(14/16/16), TG(16/16/16), TG(16/16/18), TG(16/18/18), and TG(18/18/18), which were found to be increased in the NASH group compared to NAFL patients and controls [2]. Our study also observed elevated TG with 46, 48, 50, 52, and 54 carbons, similar to the study by Chiappini et al. The main objective of the study by Ooi et al. was to investigate the lipidomic profile associated with the progression of NAFLD and the presence of NASH in patients. They analyzed alterations in the liver, visceral adipose tissue (VAT), subcutaneous adipose tissue (SAT), and plasma lipidomes of 181 patients successfully quantifying 446 lipid species across 24 classes. The liver lipidome showed considerable changes with increasing steatosis, with notable increases in triacylglycerols, diacylglycerols, and sphingolipids. Additionally, TG species, namely TG species, TG 15:0_16:0_18:1, TG 16:0_16:0_18:0, TG 16:1_16:1_16:1, TG 16:1_16:1_18:0, TG 14:0_17:0_18:1 and TG 14:0_16:0_18:1, which displayed a substantial association with steatosis progression, were found to be upregulated in the NASH group of our study as well [39]. To gain a deeper knowledge about how NAFLD affects the human liver lipidome, Vvedenskaya et al. assembled a cohort of 365 biopsies that were histologically characterized and represented the progression of NAFLD from non-steatotic obesity to overt NASH. Their study employed shotgun mass spectrometry, quantifying 316 species across 22 major lipid classes. One of the key findings was that TG 50:1 emerged as a highly specific marker of NAFLD progression, with its abundance steadily increasing from healthy obesity to NASH [29].

Dysregulations of the metabolic pathway involved in synthesis of fatty acids were highlighted in NASH. The major impact of alterations in this metabolic pathway was reinforced by the similar biochemical features (i.e., dysregulation of enzyme activities) observed in both human and animal models for this pathology, strengthening the idea that these alterations are universal in NASH. When examining the deregulated metabolic pathways in NAFLD based on the results of the targeted GC-MS analysis of 26 fatty acids, three fatty acids, namely FA 16:0, FA 16:1, and FA 18:3 n3, which belong to the long-chain fatty acid synthesis pathway, were found to be increased in NAFLD. Furthermore, the activity of enzymes involved in the elongation and desaturation of fatty acids along this metabolic pathway was studied. The activity of the enzymes was evaluated by calculating the ratio of the product to its substrate based on fatty acid analysis, which revealed that Δ9-desaturase was significantly different between healthy controls and NASH patients. Additionally, the sum of monounsaturated fatty acids was higher in the liver tissue of NASH patients.

Increased circulating fatty acids could be an important cause of hepatic lipotoxicity. More specifically, liver damage has been specifically attributed to the toxic effects of SFA accumulation particularly for the FA 16:0. In vitro studies have shown that SFAs induce the synthesis of pro-inflammatory cytokines, leading to apoptosis and disruption of insulin signalling [40]. Excessive accumulation of SFAs, specifically, FA 16:0 and FA 18:0 in hepatocytes, is capable of stressing the endoplasmic reticulum and is a major cause of damage [41]. In addition to SFAs, MUFAs were also elevated in liver tissue samples during the development and progression of NAFLD [42]. Significantly higher concentrations of palmitoleic acid FA 16:1 n7 and oleic acid FA 18:1 n9 were found in liver biopsies [2,37] and in liver tissue of muscle mice models with NAFLD [42], and interestingly, increased ratios of FA 16:1n7/FA 16:0 and FA 18:1n9/FA 18:0 in patients with NAFLD [2,37,43], suggesting an increase in Δ9-desaturase activity during disease development [37,43].

Although the total number of samples collected in this study was limited, our results align with the findings of former research studies, indicating the high accuracy of the current lipid species in classifying the patients into the respective study groups. An important point is that although the analyses were conducted using a minuscule tissue weight, the obtained results were valuable and precise, capable of classifying disease stages. This precision is noteworthy, as it demonstrates the efficacy of the state-of-the-art analytical techniques employed. The absence of data regarding participants’ long-term dietary habits needs also to be acknowledged. Overall, even though the optimal set of features for the model used to predict the disease and its activity may show variations depending on the population studied and its specific characteristics, the disease lipid pattern has certain stable characteristics. The limitation of our study is the relatively small sample size, which resulted in notable differences in certain parameters, such as gender. To prove this rationale, a thorough evaluation process involving a large and diverse group of participants from multiple centers to validate the diagnostic potential of this biomarker panel is deemed necessary.

## 4. Materials and Methods

### 4.1. Study Design

The present study was a case–control trial that included three distinct groups of individuals: patients with verified NAFLD, including both NAFL and NASH, as well as a control group comprising healthy individuals related to the disease, as determined by the histological findings. The study’s criteria have been detailed in an earlier publication by our group [33]. Liver biopsies were obtained from 18 individuals diagnosed with NAFL (n = 5) and NASH (n = 7), while healthy controls (n = 6) were acquired during laparoscopic cholecystectomy. The biopsies were collected after overnight fasting and a homogeneous low-fat diet for the past 24 h, flushed with saline to remove excess blood, and immediately stored at −80 °C until the analysis. Additionally, blood samples were taken from all participants for the analysis of biochemical markers. All participants were recruited for the study between June 2021 and June 2023 after providing written informed consent. The universally accepted disease criteria were used for the disease diagnosis and further classification into the study groups [5]. The research adhered to the principles outlined in the Declaration of Helsinki [44], received approval from the Institutional Review Board of the Medical School of Aristotle University of Thessaloniki, and underwent scrutiny and approval by the Bioethics Board of the Medical School of Aristotle University of Thessaloniki, with the assigned protocol number being 4.399/26/01/2021. In this study, the participants met both the old and the new nomenclature criteria; however, as the study population was recruited before the establishment of the new MASLD nomenclature, the formerly used terms of NAFLD, NASH, NAFL are used in the current manuscript for the description of the study groups.

### 4.2. Chemicals and Materials

Methanol (MeOH), acetonitrile (MeCN) and methyl-tert-butyl-ether (MTBE; ≥99%) and formic acid (all ULC/MS-CC/SFC grade) were obtained from CHEM-LAB NV (Zedelgem, Belgium). Isopropanol (i-PrOH) was purchased from Fisher Scientific (International Inc., Hampton, NH, USA). Ammonium formate (NH_4_HCO_2_; MS grade) and 2,6-Di-tert-butyl-4-methylphenol (BHT) were obtained from Sigma-Aldrich (Merck, Darmstadt, Germany). Deionized water (ddH_2_O) was ultrapurified by a Millipore (Bedford, MA, USA) instrument delivering water quality of a resistivity ≥ 18.2 MΩ∙cm. SPLASH^®^ LIPIDOMIX^®^ was purchased from Avanti Polar Lipids (Avanti Polar Lipids, Inc., Alabaster, AL, USA) and nonadecanoic acid (FA 19:0) was obtained from Sigma-Aldrich (Merck, Darmstadt, Germany).

### 4.3. Liver Lipidomics and Fatty Acid Analysis

Liver biopsies (0.39–6.36 mg) were transferred to 1.5 mL tubes containing 1.0 mm ceramic beads. A mixture of the organic extraction solvent MTBE:MeOH 3:1 (*v*/*v*), containing 0.01% (*w*/*v*) BHT, was added to the bead beating tubes containing the weighed tissue, with the amount of solvent added being proportional to the tissue weight. Specifically, in 1 mg of the tissue, 1 μL of the internal standard SPLASH^®^ LIPIDOMIX^®^ and 300 μL of the solvent mixture were added. Homogenization was followed by performing 4 cycles for 30 s, with a speed of 6.00 m/s, using a Bead mill Homogenizer (BEAD RUPTOR ELITE, Omni International, Kennesaw, GA, USA). After homogenization, the samples were incubated for 10 min in −20 °C and were centrifuged for 30 min at 4 °C at 11,180× *g*. In total, 450 µL of the supernatant was transferred to a 1.5 mL Eppendorf tube and evaporated to dryness in vacuo (SpeedVac, Eppendorf Austria GmbH, Wien, Austria), followed by reconstitution in 200 μL of i-PrOH. The exact extraction solvent’s volumes are provided in Table S3. A pooled sample (Quality Control Sample, QC) was prepared as a representative by mixing equal volumes of each supernatant, respectively. Group-specific QC samples for the control, NAFL and NASH were prepared as well. Diluted QCs (1:2, 1:4, 1:6, 1:8 in i-PrOH) were also analyzed to evaluate the dilution integrity of the detected features. The samples were analyzed in a randomized order, with QC samples being analyzed every 5 individual samples, resulting in a total of 5 QC samples being analyzed. Initially, blank samples, procedural blank samples, 4 QCs for equilibration and diluted QCs were analyzed. In addition, group-specific QCs were also injected in positive and negative modes, followed by the analysis of individual samples in both ionization modes.

For fatty acid analysis, in 100 μL of the supernatant, 10 μL of nonadecanoic acid (FA 19:0, 100 μg/mL) was added and evaporated to dryness in vacuo (SpeedVac, Eppendorf Austria GmbH, Wien, Austria). The dried extracts were reconstituted in 750 µL MeOH and 150 µL MeOH 8% HCl followed by acidic esterification at 80 °C for 1 h, as described in [45]. The fatty acid methyl esters were extracted with 500 μL of hexane, vortexed for 10 min and centrifuged for 10 min at 11,180× *g*. In total, 300 μL of the supernatant was evaporated to dryness in vacuo and the samples were preconcentrated to 50 μL of hexane. A pooled sample (Quality Control Sample, QC) was prepared as a representative by mixing equal volumes of each supernatant, respectively, and the samples were analyzed in randomized order.

### 4.4. Instrumentation for Untargeted Lipidomics and Fatty Acid Analyses

For sample analysis, a UHPLC Elute system equipped with an Elute autosampler was used. The autosampler vial tray was maintained at 8 °C. An Acquity UPLC CSH C18, 2.1 × 100 mm, 1.7 μm column (Waters Ltd., Elstree, UK), equipped with a pre-column Acquity UPLC CSH C18Van-Guard (Waters Ltd., Elstree, UK), was used for chromatographic separation. The column temperature was maintained at 50 °C and the flow rate was set at 0.3 mL/min during the analysis. A 30 min gradient mobile phase system was employed: the mobile phase A consisted of ACN/H_2_O 50:50 (*v*/*v*) and mobile phase B of i-PrOH/ACN/H_2_O 85:10:5 (*v*/*v*/*v*), with both containing 5 mM ammonium formate and 0.1% formic acid (FA). The gradient profile was as follows: 0–20 min—10 to 86% B, 20–22 min—86 to 95% B, 22–26 min—95% B isocratic, 26–26.1 min—95 to 10% B and 26.1–34.0 min—10% B isocratic for column re-equilibration. The needle was washed with the strong wash solvent i-PrOH/ACN/MeOH/H_2_O 30:30:30:10 (*v*/*v*/*v*/*v*) (2500 μL) and weak wash solvent ACN/H_2_O 60:40 (*v*/*v*) (1000 μL) before and after each injection. The MS data were acquired using a TIMS TOF mass spectrometer (Bruker, Billerica, MA, USA) in positive and negative ionization modes. Data-dependent acquisition (DDA) was performed to enhance the annotation procedure. In the ESI source, the end plate offset was set to 500 V, while the capillary voltage was set ±4.5 kV for positive and negative modes, respectively. The nitrogen as the dry gas was run at the rate of 10 L/min at a dry temperature of 200 °C. The nebulizer gas was set at 2.0 bar. The peak detection threshold was set to 100 counts. In DDA analysis, auto MS/MS was applied using dynamic MS/MS spectra acquisition with 6 and 10 Hz as the minimum and maximum spectra rates, respectively. Collision energy was set at 20 V for precursor ions below 100 *m*/*z*, 30 V for precursor ions with *m*/*z* that ranged from 100 to 1000 and 40 V for precursor ions with *m*/*z* thatranged from 1000 to 2000 *m*/*z*. Calibrant (sodium formate, 10 mM) was infused to MS during 0.1–0.3 s with a flow rate of 10.0 µL/h [46].

Fatty acid analysis was performed on an Agilent Technologies 8860 GC, combined with a 5977 MSD (Agilent Technologies, Santa Clara, CA, USA). Fatty acid methyl esters were separated on an Agilent 100 m HP-88 column (0.25 μm, i.d. of 0.25 μm). In total, 26 fatty acid methyl esters were quantified, namely capric acid (FA 10:0), lauric (FA 12:0), tridecanoic acid (FA 13:0), myristic (FA 14:0), pentadecanoic (FA 15:0), palmitic (FA 16:0), heptadecanoic (FA 17:0), stearic (FA 18:0), arachidic (FA 20:0), heneicosanoic acid (FA 21:0), behenic (FA 22:0), tricosanoic acid (FA 23:0), lignoceric (FA 24:0), tetradecenoic acid (FA 14:1), palmitoleic (FA 16:1), cis-9-Oleic (FA 18:1), cis-11-Eicosenoic (FA 20:1), nervonic (FA 24:1), linoleic (FA 18:2), verniceic acid (FA 20:2), gamma linolenic (FA 18:3n6), alpha linolenic (FA 18:3n3), dihomogamma linolenic (FA 20:3n6), arachidonic (FA 20:4n6), cis-5,8,11,14,17 eicosapentaenoic (FA 20:5n3) and cis-4,7,10,13,16,19 docosahexaenoic (FA 22:6n3) acids.

### 4.5. Identification and Quantification of Lipid Species

The lipid species from the untargeted lipidomics analysis were identified using Lipostar2 (version 2.0.2 Molecular Discovery Ltd., Hertfordshire, UK) using the LIPID MAPS structure database (version September 2021) [47]. The raw data files from QC and group-specific QC samples acquired in positive and negative ionization modes were imported directly to the software and aligned using the default settings. The Savitzky–Golay algorithm was employed for automated peak detection, with the following settings: a window size of 7, a polynomial degree of 2, one iteration of multi-pass, and a minimum signal-to-noise ratio of 3. Mass tolerance settings were set to 10 ppm with RT tolerance 0.2 min. The filters of retain lipids with isotopic patterns and retain lipids with MS/MS were applied. The following parameters were used for lipid identification: 5 ppm precursor ion mass tolerance and 20 ppm product ion mass tolerance. The automatic approval was performed to keep structures with a quality of 3–4 stars. To confirm the accuracy of lipid annotations, retention times of a given lipid species against their Kendrick mass defect to the hydrogen base were plotted using an in-house script in Python programming language [48,49]. The peak area integration of sphingolipids, phospholipids, and glycerolipids was performed via Skyline v.21.1.0.146 (MacCoss Lab) [50], where the peak boundaries were defined, manually adjusted, and verified. Data from fatty acids analysis were processed by MassHunter Workstation (Version 10.0) software.

### 4.6. Data Analysis and Visualization

Univariate statistical analysis was conducted in Python programming language and GraphPad Prism v8.0.1 software. Continuous data in baseline characteristics are presented as medians ± standard deviation (SD), while categorical data are presented as frequencies (N) with percentages (N%). The Shapiro–Wilk test was employed for normal distribution evaluation. A one-way ANOVA was conducted for normally distributed lipids followed by Bonferroni adjustment, while the Kruskal–Wallis test was used for non-normally distributed lipids. Post hoc Dunn’s test was applied when the *p*-value was less than 0.05. The chi-square test was utilized for categorical parameters. Statistical significance was defined as a value of *p*-value < 0.05. SIMCA 13.0.3 (UMETRICS AB, Umea, Sweden) software [51] was used for multivariate analysis such as unsupervised principal component analysis (PCA), partial least squares analysis (PLS) and orthogonal partial least squares discriminant analysis (OPLS-DA). The identification of significant lipids was performed using the filter S-plot with an absolute *p*-value > |0.05| and p(corr) > |0.5|, respectively. To assess the model quality, parameters such as the goodness of fit in the X (R^2^X) and Y (R^2^Y) variables, as well as predictability (Q^2^YCV), were assessed through the software. A *p*-value from CV ANOVA analysis indicating the statistical significance of the model was calculated via the software as well. Pareto scaling was used in all models. Following this, ANCOVA analysis was conducted to account for waist circumference and HOMA-IR differences between groups, significant parameters affecting intra-abdominal fat and insulin resistance, respectively. Non-normally distributed features were logarithmically transformed before the ANCOVA analysis. An adjusted *p*-value < 0.05 was considered significant. To apply the strictest criteria for selecting significant lipids, only those lipids that were found significant in both multivariate and univariate analyses, with a *p*-value < 0.05 before and after the ANCOVA analysis, were considered statistically significant.

## 5. Conclusions

This study utilizes a detailed lipidomic approach, focusing on intact lipids and fatty acids, and investigates these species directly in liver biopsy tissue extracts from both NAFLD patients and controls using advanced analytical techniques. Our findings indicate that patients with NASH have a distinct liver lipid profile that discriminates them from NAFL patients and controls, based on observed changes in triglyceride species and fatty acids such as FA 16:0, FA 16:1, FA 18:3 n6, the total of MUFA, and the Δ9-desaturase activity ratio. This research enhances our understanding of how disrupted lipid metabolism contributes to the progression of NAFLD, corroborating findings from previous studies. However, given the small sample size of this study, larger-scale research is needed to confirm these findings. Future research into lipid metabolism could refine risk assessment methods, especially considering the current limited knowledge about the progression from steatosis to NASH, and could have significant clinical, diagnostic, and therapeutic implications.

## Figures and Tables

**Table 1:** Baseline characteristics of the study population and comparison between the study groups.

Parameters	Total Population	Control (n = 6)	NAFL (n = 5)	NASH (n = 7)	*p*-Value Control-NAFL	*p*-Value Control-NASH	*p*-Value NAFL-NASH
**Demographics and clinical characteristics**							
Gender (Male)	8 (44.4%)	0 (0%)	3 (37.5%)	5 (62.5%)	0.1380	**0.0413**	0.9999
Age (years)	54 ± 10	49 ± 10	57 ± 8	56 ± 11	0.7055	0.7925	0.9999
BMI (kg/m^2^)	29.3 ± 4.9	25.7 ± 5.3	28.9 ± 4.4	32.2 ± 3.5	0.8113	0.0657	0.6061
Diabetes Mellitus	7 (38.8%)	1 (14.2%)	1 (14.2%)	5 (71.6%)	0.9999	0.2396	0.2396
Arterial Hypertension	8 (44.4%)	1 (12.5%)	3 (37.5%)	4 (50.0%)	0.7192	0.7152	0.9999
Metabolic Syndrome	9 (50.0%)	1 (11.1%)	2 (22.2%)	6 (66.7%)	0.9999	0.0788	0.3178
Waist Circumference (cm)	104 ± 17.6	89.2 ± 13.9	105 ± 18	115 ± 11.9	0.3172	**0.0212**	0.6604
HOMA-IR	3.50 ± 4.70	1.70 ± 1.00	1.90 ± 1.30	8.80 ± 4.70	0.9999	**0.0134**	**0.0108**
**Biochemical parameters**							
ALT (U/L)	26.0 ± 45.1	26.0 ± 16.8	23.0 ± 10.0	53.0 ± 58.9	0.9999	0.1284	0.1089
AST (U/L)	28.0 ± 30.5	22.0 ± 10.1	23.0 ± 4.10	46.0 ± 39.1	0.9999	**0.0270**	**0.0146**
GGT (U/L)	28.0 ± 185	12.0 ± 21.8	20.0 ± 5.80	79.0 ± 269	0.9999	0.1187	0.2563
ALP (U/L)	78.7 ± 29.8	84.4 ± 30.2	66.2 ± 22.4	83.5 ± 35.2	0.6246	0.9988	0.6071
Insulin (μLU/mL)	16.2 ± 10.3	8.80± 5.60	8.80 ± 5.80	26.7 ± 4.30	0.9999	**0.0001**	**0.0001**
Platelets (×10^3^) (K/μL)	224 ± 75.8	268 ± 93.3	218 ± 72.4	197 ± 59.7	0.5553	0.2664	0.8779
HbA1c (%)	5.60 ± 1.70	5.40 ± 0.40	5.40 ± 0.30	6.90 ± 2.30	0.9999	0.2246	0.1109
FBG (mg/dL)	91.0 ± 49.6	89.0 ± 15.8	83.0 ± 18.5	110 ± 69.0	0.9999	0.6740	0.4691
Total Cholesterol (mg/dL)	185 ± 48.9	195 ± 34.9	155 ± 53.5	200 ± 50.5	0.5880	0.9999	0.3742
LDL-c (mg/dL)	103 ± 37.4	116 ± 37.4	86.6 ± 44.0	106 ± 33.5	0.6918	0.9999	0.9999
HDL-c (mg/dL)	51.1 ± 13.9	62.2 ± 17.3	45.2 ± 11.6	47.4 ± 8.8	0.1525	0.1942	0.9999
Total Triglycerides (mg/dL)	124 ± 144	86.0 ± 25.6	124 ± 30.3	213 ± 179	0.9999	**0.0128**	0.1686
Ferritin (ng/mL)	165 ± 320	76.0 ± 20.2	171 ± 100	218 ± 446	0.3986	0.0660	0.9999
Uric Acid (mg/dL)	5.24 ± 1.5	4.10 ± 1.1	4.86 ± 0.80	6.33 ± 1.40	0.9334	**0.0149**	0.1370
Albumin (gr/dL)	4.43 ± 0.30	4.52 ± 0.20	4.44 ± 0.44	4.34 ± 0.22	0.9019	0.5815	0.8517
**Histological characteristics and scores in liver biopsies**							
NAS	2.0 ± 2.40	0 ± 0	2.0 ± 0.44	5.0 ± 0.75	0.3266	**0.0004**	0.1131
NFS	−1.3 ± 1.9	−2.1 ± 1.8	−1.4 ± 1.9	−0.3 ± 1.4	0.5721	0.0761	0.9797
FIB-4	1.3 ± 0.7	0.9 ± 0.5	1.2 ± 0.7	2.3 ± 0.6	0.619	**0.0295**	0.428
Steatosis	1.0 ± 1.0	0 ± 0	1.0 ± 0	2.0 ± 0.5	0.3108	**0.0003**	0.1047
Balloning	0 ± 0.8	0 ± 0	0 ± 0	1.0 ± 0.5	0.9999	**0.0034**	**0.0034**
Inflammation	1 ± 0.8	0 ± 0	1.0 ± 0.4	2.0 ± 0.5	0.2459	**0.0015**	0.3254
Fibrosis	0 ± 1.4	0 ± 0	0 ± 0.4	2.0 ± 1.6	0.9999	**0.012**	0.0514

Continuous variables are presented as medians ± SD. Categorical parameters are presented as counts (N) and percentage (N%) for each parameter’s category. A one-way ANOVA and Kruskal–Wallis tests were conducted for normally and non-normally distributed continuous parameters, respectively, while Chi square (χ^2^) test was conducted for the categorical variables, in order to assess the statistical significance of the comparison between the two distinct NAFLD groups and controls. The threshold for statistical significance was set at *p* < 0.05. Abbreviations: BMI: Body Mass Index, HDL-c: high-density lipoprotein cholesterol, LDL-c: low-density lipoprotein cholesterol, ALT: alanine transaminase, AST: aspartate aminotransferase, GGT: gamma-glutamyl transferase, ALP: alkaline phosphatase, FBG: fasting plasma glucose, NAS: NAFLD Activity Score, NFS: NAFLD Fibrosis Score; FIB-4: Fibrosis-4. *p* values of the statistically significant parameters are bolded.

**Table 2:** Statistically significant lipids in liver biopsies that contribute to the discrimination between the healthy controls and patients with NASH and NAFL–NASH patients. *p* values, adjusted p* values, Log2FC, CV%, and VIP scores are presented for the TG species. Bolded values correspond to *p* ≤ 0.05.

Lipids	Lipid Species	Adjusted p* Values	Controls–NASH			NAFL–NASH			
			*p* Value	VIP	Log2FC	*p* Value	VIP	Log2FC	CV%
TG 42:0	TG 10:0_16:0_16:0 TG 12:0_14:0_16:0 TG 14:0_14:0_14:0	**2.50 × 10^−2^**	**7.10 × 10^−3^**	0.8	3.17	**2.10 × 10^−2^**	0.9	3.55	3.79
TG 42:1	TG 10:0_14:0_18:1 TG 10:0_16:0_16:1 TG 12:0_12:0_18:1 TG 12:0_14:0_16:1 TG 12:0_14:1_16:0 TG 14:0_14:0_14:1 TG 8:0_16:0_18:1	**7.20 × 10^−3^**	**3.40 × 10^−2^**	0.7	2.37	**3.10 × 10^−3^**	0.9	3.42	4.23
TG 44:0	TG 12:0_16:0_16:0 TG 14:0_14:0_16:0	**5.80 × 10^−3^**	**3.30 × 10^−3^**	1.1	3.04	**4.30 × 10^−2^**	1.3	3.1	3.55
TG 44:1	TG 10:0_16:0_18:1 TG 12:0_14:0_18:1 TG 12:0_16:0_16:1 TG 14:0_14:0_16:1	**9.50 × 10^−3^**	**3.30 × 10^−3^**	1.4	3.21	**4.30 × 10^−2^**	1.6	3.62	3.07
TG 44:2	TG 10:0_16:0_18:2 TG 10:0_16:1_18:1 TG 12:0_14:0_18:2 TG 12:0_14:1_18:1 TG 12:0_16:0_16:2 TG 12:0_16:1_16:1 TG 14:0_14:1_16:1 TG 14:1_14:1_16:0 TG 8:0_18:1_18:1	**3.00 × 10^−2^**	**5.80 × 10^−3^**	0.9	2.91	**4.10 × 10^−2^**	1.1	3.57	3.67
TG 46:0	TG 14:0_14:0_18:0 TG 14:0_16:0_16:0	**3.30 × 10^−2^**	**3.30 × 10^−3^**	1	1.96	**4.30 × 10^−2^**	1.2	2.03	1.68
TG 46:1	TG 12:0_16:0_18:1 TG 14:0_14:0_18:1 TG 14:0_16:0_16:1	**3.30 × 10^−5^**	**2.30 × 10^−3^**	2.3	3.32	**3.80 × 10^−2^**	2.6	2.84	2.42
TG 46:2	TG 10:0_18:1_18:1 TG 12:0_16:0_18:2 TG 12:0_16:1_18:1 TG 14:0_14:0_18:2 TG 14:0_14:1_18:1 TG 14:0_16:1_16:1 TG 14:1_16:0_16:1	**7.10 × 10^−3^**	**2.80 × 10^−3^**	1.8	3.28	**3.20 × 10^−2^**	2.1	3.41	3.12
TG 46:3	TG 10:0_18:1_18:2 TG 12:0_16:1_18:2 TG 14:0_14:1_18:2 TG 14:1_14:1_18:1 TG 14:1_16:1_16:1	**1.40 × 10^−2^**	**3.40 × 10^−3^**	1	3.18	**2.60 × 10^−2^**	1.1	3.47	3.79
TG 48:0	TG 14:0_16:0_18:0	**2.80 × 10^−2^**	**2.30 × 10^−3^**	1	1.86	**3.80 × 10^−2^**	1.2	1.69	1.77
TG 48:1	TG 14:0_16:0_18:1 TG 14:0_16:1_18:0 TG 15:0_15:0_18:1 TG 15:0_16:1_17:0 TG 16:0_16:0_16:1	**2.30 × 10^−5^**	**9.90 × 10^−4^**	2.5	2.54	7.50 × 10^−2^	2.6	1.6	1.8
TG 48:2	TG 12:0_18:1_18:1 TG 14:0_16:0_18:2 TG 14:0_16:1_18:1 TG 14:1_16:0_18:1 TG 16:0_16:0_16:2 TG 16:0_16:1_16:1	**7.40 × 10^−7^**	**1.50 × 10^−3^**	2.8	2.97	**5.40 × 10^−2^**	3	2.1	1.15
TG 48:3	TG 12:1_18:1_18:1 TG 14:0_16:1_18:2 TG 14:1_16:0_18:2 TG 14:1_16:1_18:1 TG 16:1_16:1_16:1	**3.00 × 10^−3^**	**2.80 × 10^−3^**	2.2	3.65	**3.20 × 10^−2^**	2.5	3.02	4.07
TG 48:4	TG 12:0_18:2_18:2 TG 14:1_16:1_18:2	**9.90 × 10^−3^**	**5.00 × 10^−3^**	1.1	3.89	**1.80 × 10^−2^**	1.2	3.08	3.35
TG 49:0	TG 15:0_16:0_18:0 TG 15:0_17:0_17:0 TG 16:0_16:0_17:0	**3.50 × 10^−2^**	**1.90 × 10^−3^**	0.7	2.17	**4.50 × 10^−2^**	0.8	1.97	1.88
TG 49:1	TG 14:0_17:0_18:1 TG 15:0_16:0_18:1 TG 15:0_17:0_17:1 TG 16:0_16:0_17:1	**5.50 × 10^−4^**	**2.30 × 10^−3^**	1.4	2.33	**3.80 × 10^−2^**	1.6	1.84	2.82
TG 49:2	TG 13:0_18:1_18:1 TG 14:0_17:0_18:2 TG 14:0_17:1_18:1 TG 15:0_16:0_18:2 TG 15:0_16:1_18:1 TG 15:0_17:1_17:1 TG 16:1_16:1_17:0	**2.00 × 10^−3^**	**2.80 × 10^−3^**	1.1	1.85	**3.20 × 10^−2^**	1.3	1.84	3.36
TG 49:3	TG 15:0_16:1_18:2 TG 15:1_16:0_18:2 TG 15:1_16:1_18:1 TG 15:1_17:1_17:1 TG 16:1_16:1_17:1	**2.30 × 10^−2^**	**1.60 × 10^−2^**	0.6	1.82	**3.70 × 10^−2^**	0.8	2.26	2.1
TG 50:0	TG 16:0_16:0_18:0	**8.10 × 10^−3^**	**6.40 × 10^−4^**	1.4	2.76	1.00 × 10^0^	1.6	1.83	1.79
TG 50:1	TG 16:0_16:0_18:1 TG 16:0_16:1_18:0	**6.50 × 10^−5^**	**4.10 × 10^−4^**	3.3	2.29	1.40 × 10^−1^	3.4	1.37	1.11
TG 50:2	TG 14:0_18:0_18:2 TG 14:0_18:1_18:1 TG 16:0_16:0_18:2 TG 16:0_16:1_18:1 TG 16:0_16:2_18:0 TG 16:1_16:1_18:0	**2.40 × 10^−4^**	**7.90 × 10^−4^**	2.9	1.62	8.90 × 10^−2^	2.7	0.89	1.83
TG 50:3	TG 14:0_18:1_18:2 TG 16:0_16:0_18:3 TG 16:0_16:1_18:2 TG 16:0_16:2_18:1 TG 16:1_16:1_18:1	**3.20 × 10^−5^**	**1.20 × 10^−3^**	3	2.55	6.40 × 10^−2^	2.9	1.34	0.72
TG 50:4	TG 14:0_18:2_18:2 TG 14:1_16:1_20:2 TG 14:1_18:1_18:2 TG 16:1_16:1_18:2	**3.10 × 10^−3^**	**2.30 × 10^−3^**	2.4	3.92	**3.80 × 10^−2^**	2.6	2.67	3.92
TG 50:5	TG 14:0_14:0_22:5 TG 14:0_18:2_18:3 TG 14:1_18:1_18:3 TG 14:1_18:2_18:2 TG 14:2_18:1_18:2 TG 16:0_16:2_18:3 TG 16:1_16:1_18:3 TG 16:1_16:2_18:2	**8.50 × 10^−4^**	**3.40 × 10^−3^**	1	4	**2.60 × 10^−2^**	1.1	2.83	2.75
TG 51:1	TG 15:0_18:0_18:1 TG 16:0_16:0_19:1 TG 16:0_17:0_18:1	**3.00 × 10^−4^**	**9.90 × 10^−4^**	1.9	3.07	7.50 × 10^−2^	2.1	2.15	3.47
TG 51:2	TG 15:0_18:1_18:1 TG 16:0_17:1_18:1	**8.80 × 10^−6^**	**1.20 × 10^−3^**	0.9	2.28	6.40 × 10^−2^	0.9	1.3	4.61
TG 51:3	TG 16:0_17:1_18:2 TG 16:1_17:0_18:2 TG 16:1_17:1_18:1 TG 17:1_17:1_17:1	**3.10 × 10^−3^**	**1.50 × 10^−3^**	1.5	2.67	5.40 × 10^−2^	1.6	1.72	2.84
TG 52:0	TG 16:0_18:0_18:0 TG 16:0_16:0_20:0	**1.70 × 10^−3^**	**2.30 × 10^−3^**	1.1	2.57	**3.80 × 10^−2^**	1.2	2.14	3.81
TG 52:1	TG 16:0_18:0_18:1	**1.80 × 10^−4^**	**9.90 × 10^−4^**	3.8	2.97	7.50 × 10^−2^	3.9	1.56	1.53
TG 52:2	TG 16:0_18:0_18:2 TG 16:0_18:1_18:1 TG 16:1_18:0_18:1	**3.10 × 10^−3^**	**1.20 × 10^−3^**	3.7	1.2	1.00 × 10^0^	3.1	0.49	1.32
TG 52:3	TG 16:0_16:0_20:3 TG 16:0_18:0_18:3 TG 16:0_18:1_18:2 TG 16:1_18:0_18:2 TG 16:1_18:1_18:1	**1.80 × 10^−2^**	**2.10 × 10^−3^**	2.9	0.97	1.50 × 10^−1^	2.4	0.4	2.5
TG 52:4	TG 16:0_16:1_20:3 TG 16:0_18:1_18:3 TG 16:0_18:2_18:2 TG 16:1_18:1_18:2	**3.10 × 10^−3^**	**3.20 × 10^−3^**	3.5	1.76	6.90 × 10^−2^	3.2	0.85	1.14
TG 52:5	TG 16:0_18:2_18:3 TG 16:1_18:1_18:3 TG 16:1_18:2_18:2 TG 16:2_18:1_18:2	**1.20 × 10^−2^**	**2.70 × 10^−3^**	2.7	3.52	5.10 × 10^−2^	2.9	2.11	3.75
TG 53:1	TG 16:0_17:0_20:1 TG 16:0_18:0_19:1 TG 16:0_18:1_19:0 TG 17:0_17:1_19:0 TG 17:0_18:0_18:1 TG 17:1_18:0_18:0	**3.00 × 10^−2^**	**1.20 × 10^−3^**	1	3.37	6.40 × 10^−2^	1.1	2.64	2.96
TG 53:2	TG 16:0_18:1_19:1 TG 17:0_17:1_19:1 TG 17:0_18:1_18:1 TG 17:1_18:0_18:1	**1.30 × 10^−3^**	**9.90 × 10^−4^**	1.9	2.73	7.50 × 10^−2^	1.9	1.47	3.56
TG 53:3	TG 16:0_18:2_19:1 TG 16:1_18:1_19:1 TG 17:0_18:1_18:2 TG 17:1_18:0_18:2 TG 17:1_18:1_18:1	**8.30 × 10^−3^**	**2.60 × 10^−3^**	1.5	2.28	8.10 × 10^−2^	1.4	1.08	2.74
TG 54:1	TG 16:0_16:0_22:1 TG 16:0_18:0_20:1 TG 18:0_18:0_18:1	**3.30 × 10^−2^**	**1.20 × 10^−3^**	2.2	4.01	6.40 × 10^−2^	2.4	3.08	4.71
TG 54:2	TG 16:0_18:0_20:2 TG 16:0_18:1_20:1 TG 18:0_18:0_18:2 TG 18:0_18:1_18:1	**1.00 × 10^−2^**	**2.20 × 10^−3^**	3.1	2.01	9.50 × 10^−2^	2.9	0.9	1.58

Adjusted p* Values: The value is adjusted with the values of the waist circumference and the HOMA-IR values.

**Table 3:** Statistically significant liver tissue fatty acids and ratios that contribute to the discrimination between the healthy controls and patients with NASH and NAFL–NASH patient groups. *p*-values, adjusted p* values and Log2FC for each fatty acid and ratio are provided as well. *p*-values that correspond to ≤0.05 are bolded. The CV% values for FA 16:0, FA 16:1 and FA 18:3 n6 were 10.2%, 7.68% and 8.27%, respectively.

Fatty Acids and Fatty Acid Ratios	Adjusted p* Values	Control–NASH		NAFL–NASH	
		*p* Value	Log2FC	*p* Value	Log2FC
Δ9-Desaturase (FA 16:1/FA 16:0)	**4.84 × 10^−3^**	**4.79 × 10^−3^**	2.59	5.01 × 10^−2^	2.23
Δ9- Desaturase (FA 18:1/FA 18:0)	**1.37 × 10^−2^**	**1.78 × 10^−3^**	3.28	1.03 × 10^−1^	2.17
FA 16:0	**4.92 × 10^−2^**	**3.29 × 10^−2^**	1.88	**1.44 × 10^−2^**	1.79
FA 16:1	**1.52 × 10^−2^**	**7.64 × 10^−3^**	4.49	**3.40 × 10^−2^**	3.95
FA 18:3 n6	**2.90 × 10^−2^**	**6.06 × 10^−3^**	3.18	**4.14 × 10^−2^**	2.54
MUFA	**2.91 × 10^−2^**	**4.79 × 10^−3^**	0.27	**5.00 × 10^−2^**	0.32

Adjusted p* Values: The value is adjusted with the values of the waist circumference and the HOMA-IR values.

## Data Availability

Dataset is available on request from the authors.

## Supplementary Materials

The following supporting information can be downloaded at https://www.mdpi.com/article/10.3390/ijms252313067/s1.
